# Supplementary material for: CCT6A knockdown suppresses osteosarcoma cell growth and Akt pathway activation in vitro
Source: PLoS One. 2022 Dec 30;17(12):e0279851. doi: 10.1371/journal.pone.0279851 (PMC9803215; doi:10.1371/journal.pone.0279851)
Supplement: S1 Data — (PDF) [file pone.0279851.s001.pdf]

# Original data

Figure 2-5

Figure 2C

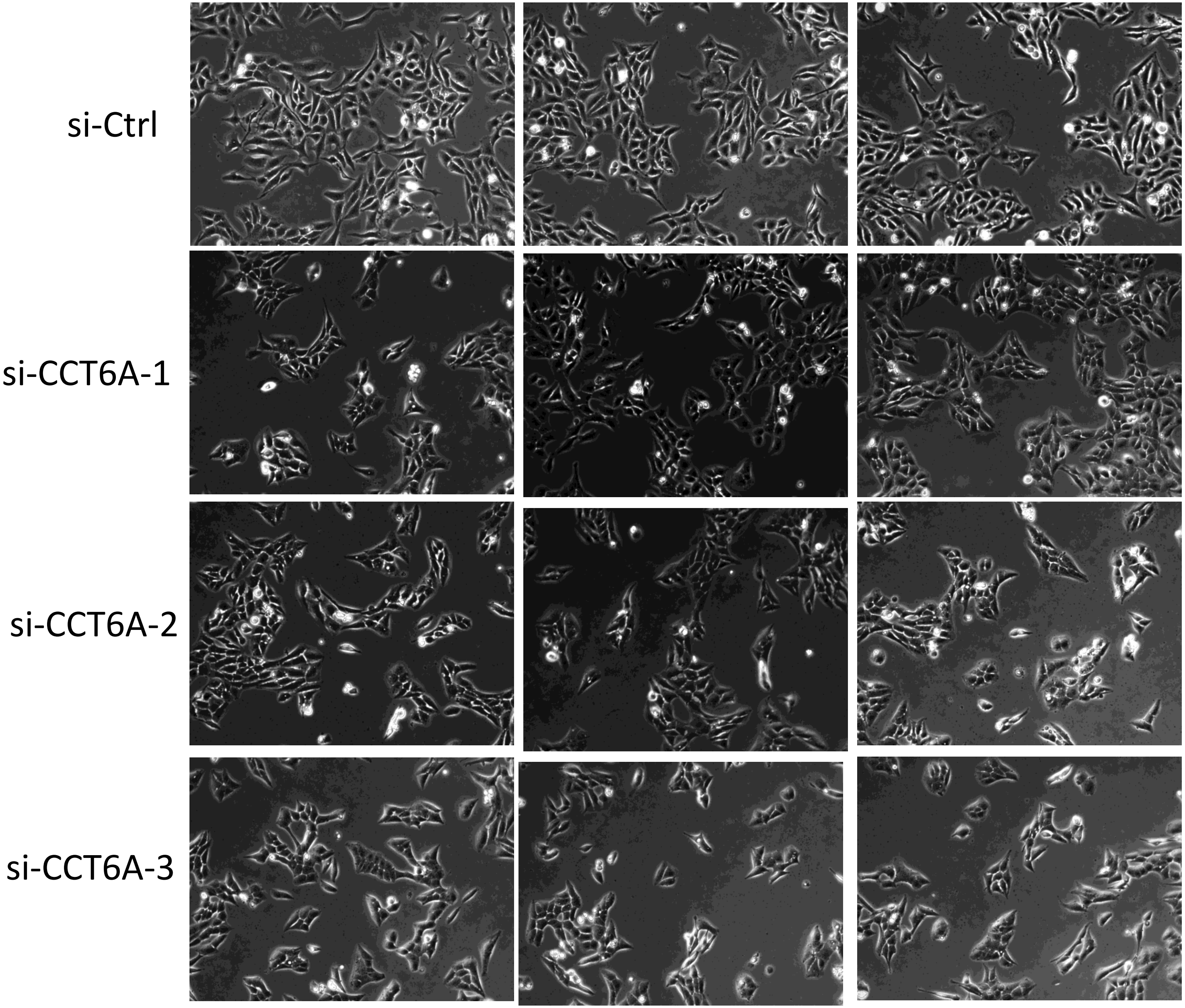

# Figure 2B and 2D

CCK-8 assay

| 24h | | | |
|---|---|---|---|
| si-Ctrl | si-CCT6A-1 | si-CCT6A-2 | si-CCT6A-3 |
| 0.404 | 0.331 | 0.332 | 0.283 |
| 0.416 | 0.343 | 0.338 | 0.301 |
| 0.381 | 0.33 | 0.309 | 0.292 |
| 0.354 | 0.358 | 0.369 | 0.298 |
| 0.438 | 0.368 | 0.331 | 0.273 |
| 0.38 | 0.354 | 0.335 | 0.324 |

| 48h | | | |
|---|---|---|---|
| si-Ctrl | si-CCT6A-1 | si-CCT6A-2 | si-CCT6A-3 |
| 0.511 | 0.438 | 0.342 | 0.253 |
| 0.495 | 0.428 | 0.386 | 0.258 |
| 0.561 | 0.457 | 0.407 | 0.272 |
| 0.562 | 0.497 | 0.409 | 0.278 |
| 0.537 | 0.444 | 0.391 | 0.292 |
| 0.596 | 0.454 | 0.421 | 0.3 |

| 72h | | | |
|---|---|---|---|
| si-Ctrl | si-CCT6A-1 | si-CCT6A-2 | si-CCT6A-3 |
| 0.839 | 0.753 | 0.543 | 0.228 |
| 0.989 | 0.738 | 0.567 | 0.228 |
| 0.895 | 0.686 | 0.565 | 0.211 |
| 0.956 | 0.744 | 0.537 | 0.233 |
| 0.928 | 0.717 | 0.566 | 0.232 |
| 0.932 | 0.684 | 0.537 | 0.23 |

cell counts assay

| | | | |
|---|---|---|---|
| si-Ctrl | $10.7*10^{5}$ | $10.2*10^{5}$ | $10.3*10^{5}$ |
| si-CCT6A-1 | $8.94*10^{5}$ | $8.29*10^{5}$ | $10.3*10^{5}$ |
| si-CCT6A-2 | $5.6*10^{5}$ | $5.5*10^{5}$ | $6.46*10^{5}$ |
| si-CCT6A-3 | $1.7*10^{5}$ | $2.08*10^{5}$ | $1.63*10^{5}$ |

Figure 3

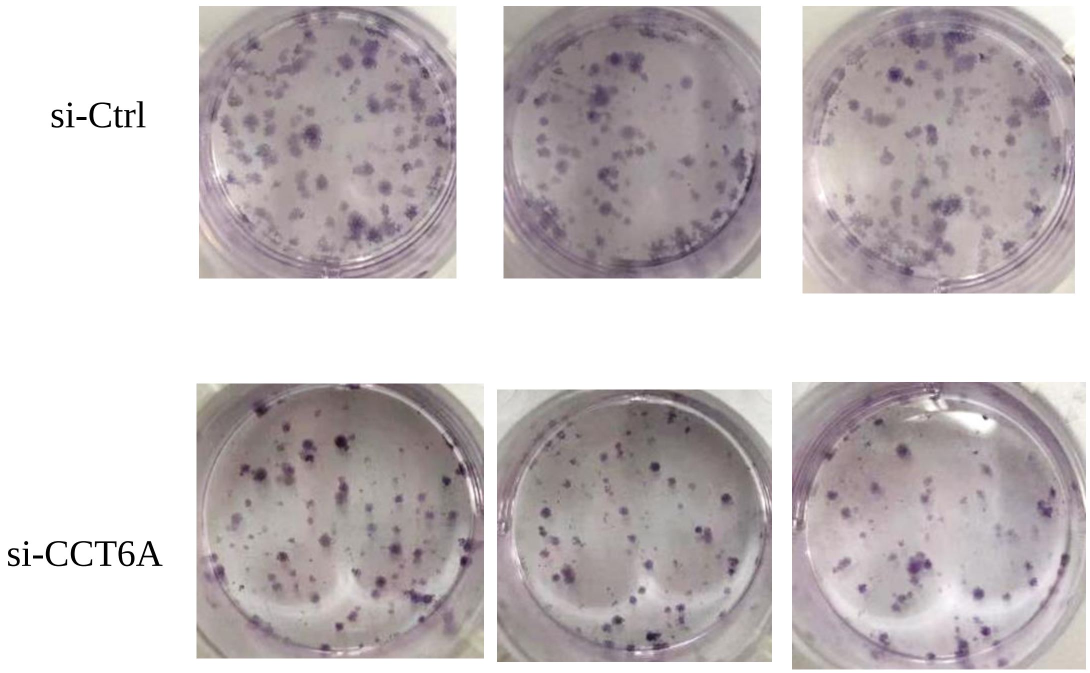

# Figure 4

si-Ctrl

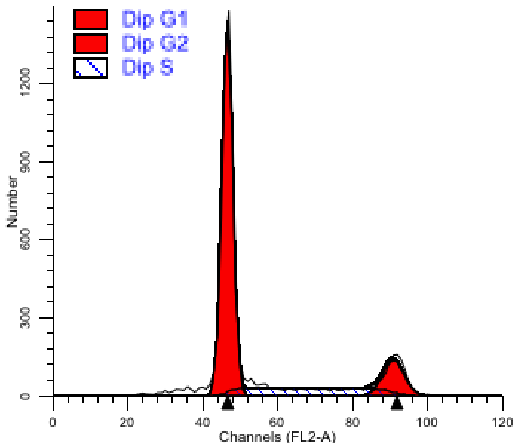


Dip G1: 71.14 % Dip G2: 12.80 %
Dip S: 16.07 %

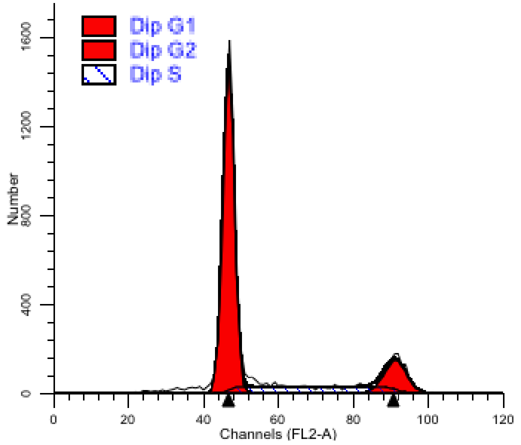


Dip G1: 70.26 % Dip G2: 13.08 %
Dip S: 16.66 %

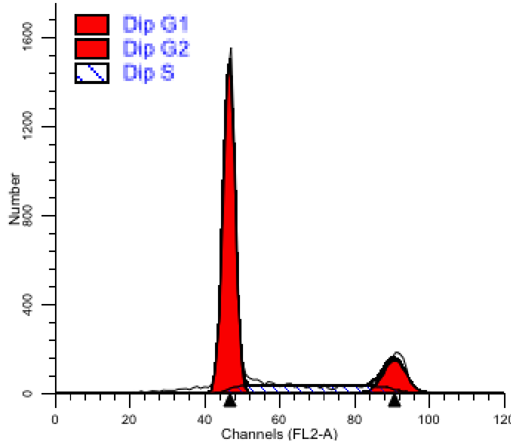


Dip G1: 70.18 % Dip G2: 13.00 %
Dip S: 16.82 %

si-CCT6A

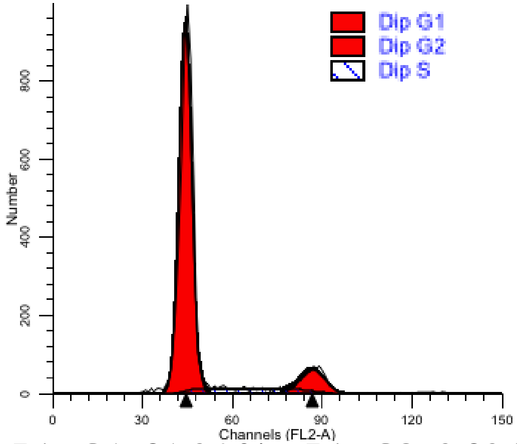


Dip G1: 81.94 % Dip G2: 9.89 %
Dip S: 8.17 %

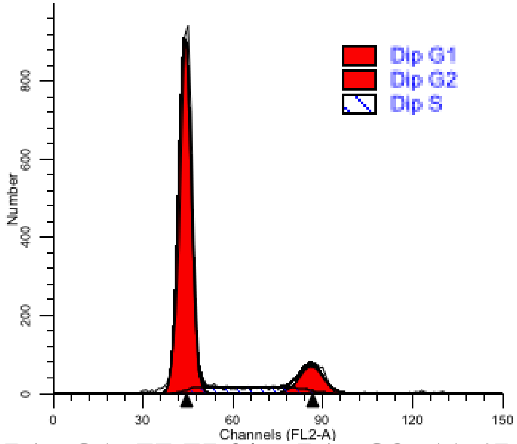


Dip G1: 77.75 % Dip G2: 11.45 %
Dip S: 10.80 %

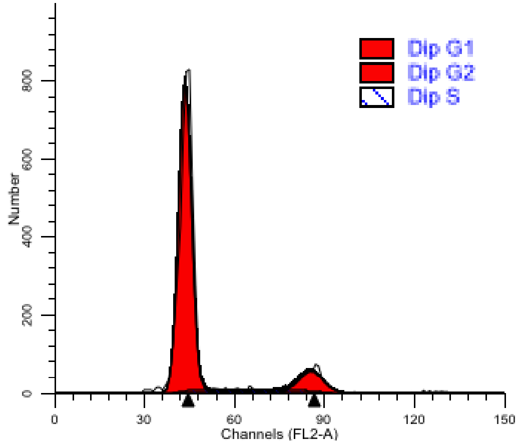


Dip G1: 81.79 % Dip G2: 10.99 %
Dip S: 7.22 %

# Figure 5

si-Ctrl

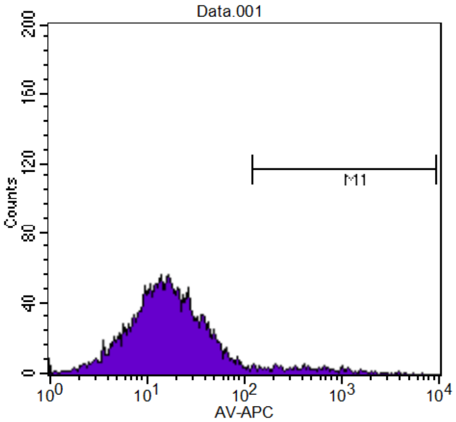


Apoptosis rate (M1):5.14%

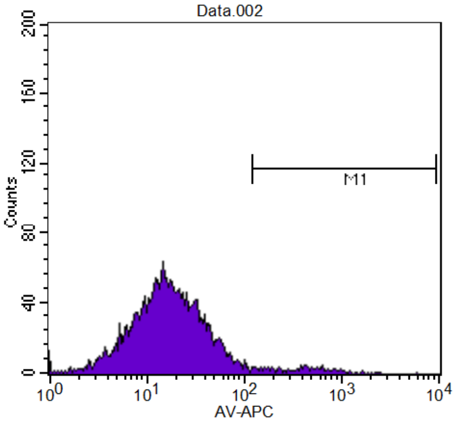


Apoptosis rate (M1): 3.74%

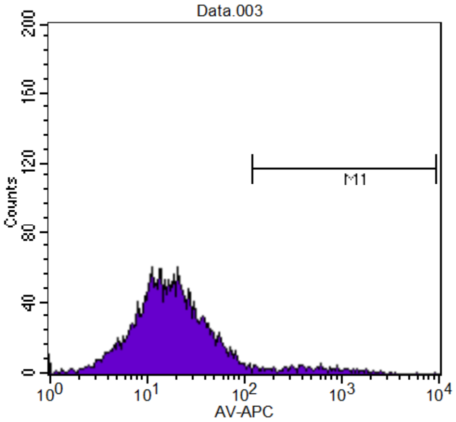


Apoptosis rate (M1): 4.63%

si-CCT6A

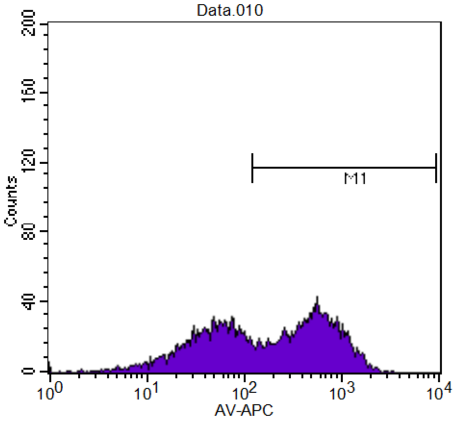


Apoptosis rate (M1): 56.03%

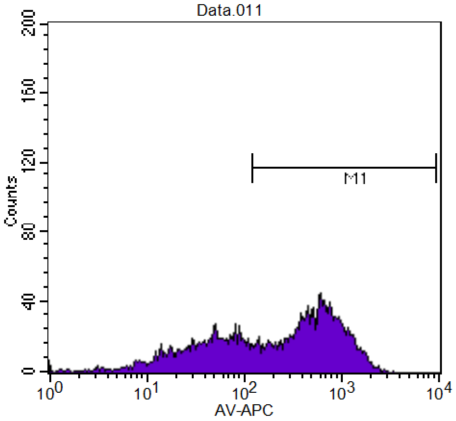


Apoptosis rate (M1): 62.03%

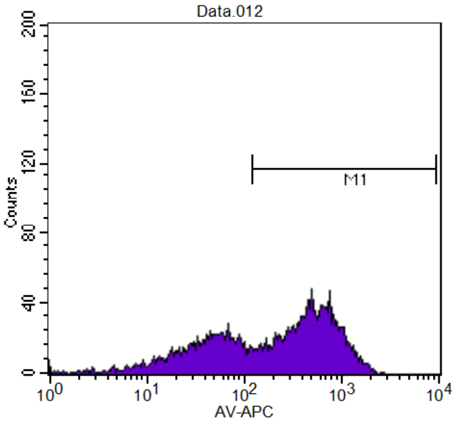


Apoptosis rate (M1): 61.72%
