# Supplementary figures and images for: CCT6A knockdown suppresses osteosarcoma cell growth and Akt pathway activation in vitro
Source: PLoS One. 2022 Dec 30;17(12):e0279851. doi: 10.1371/journal.pone.0279851 (PMC9803215; doi:10.1371/journal.pone.0279851)

# Western blot

Original data

Figure2a

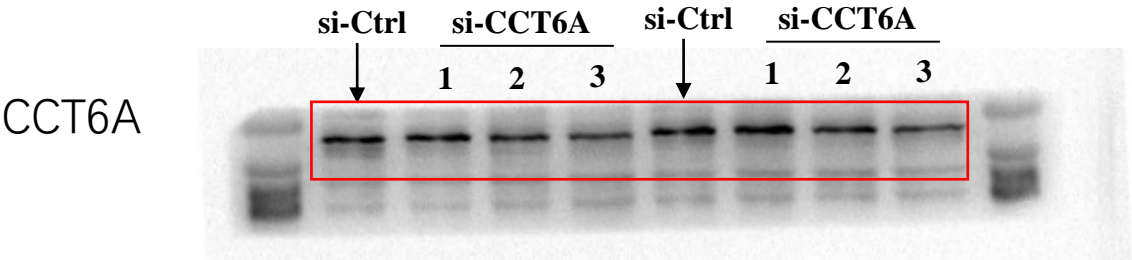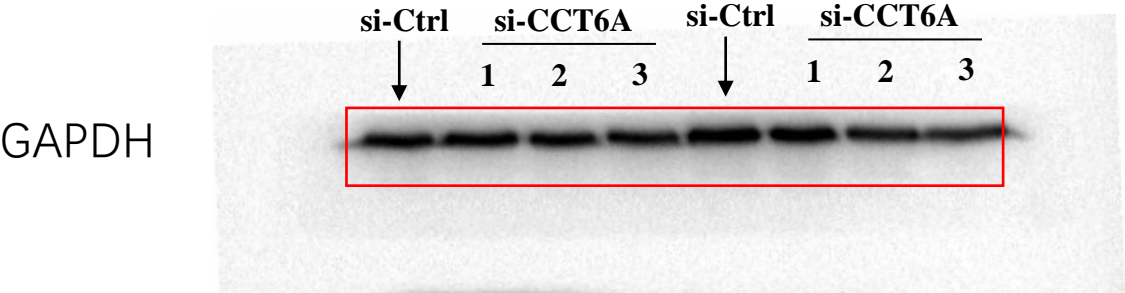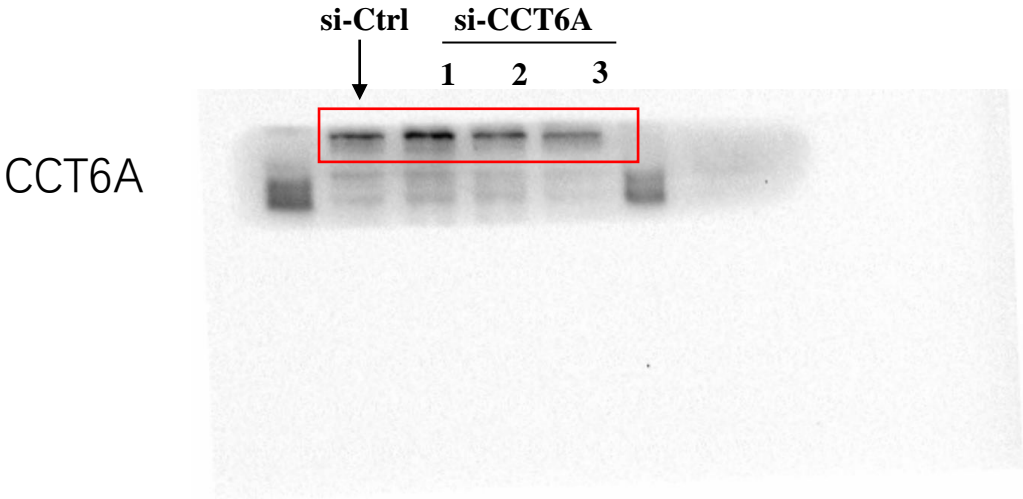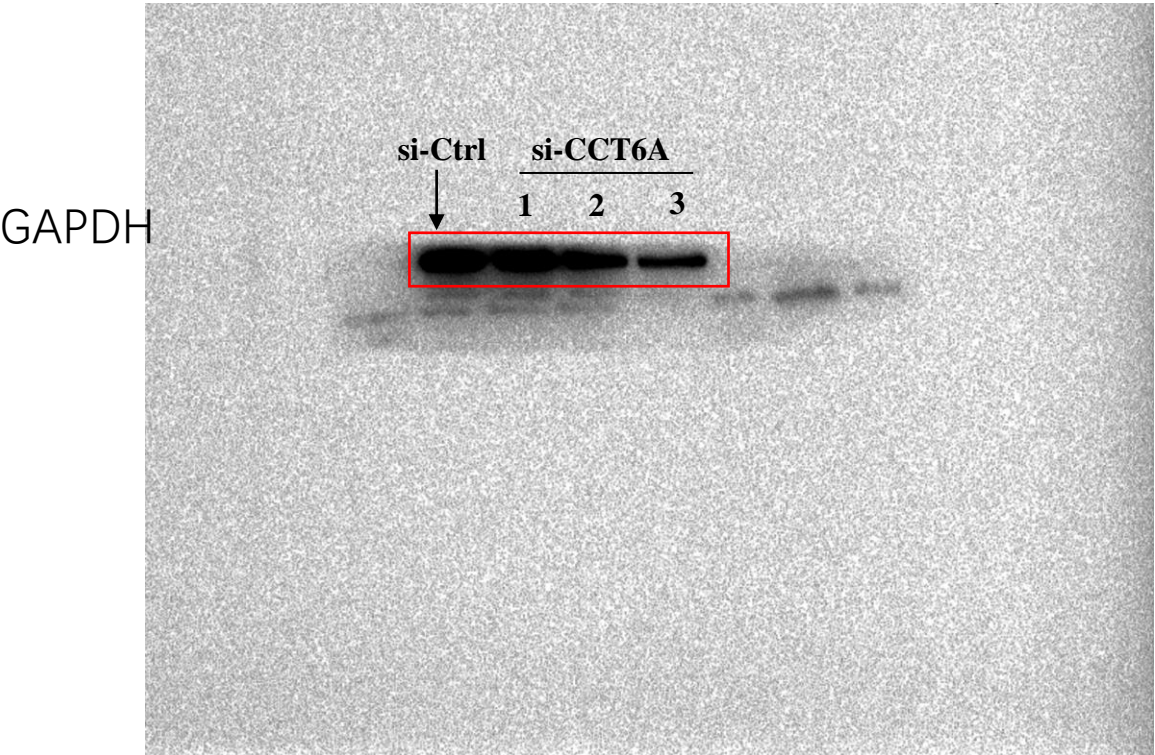

Figure 6

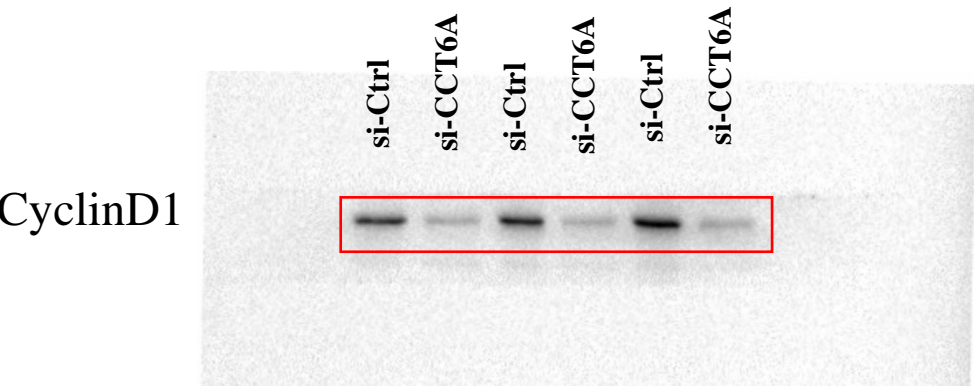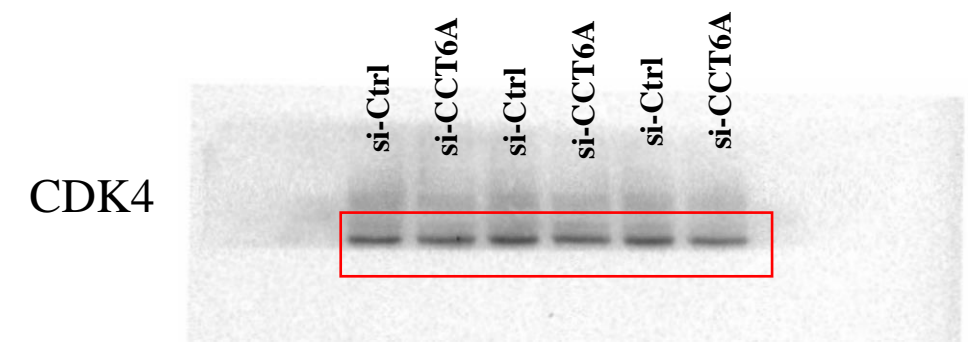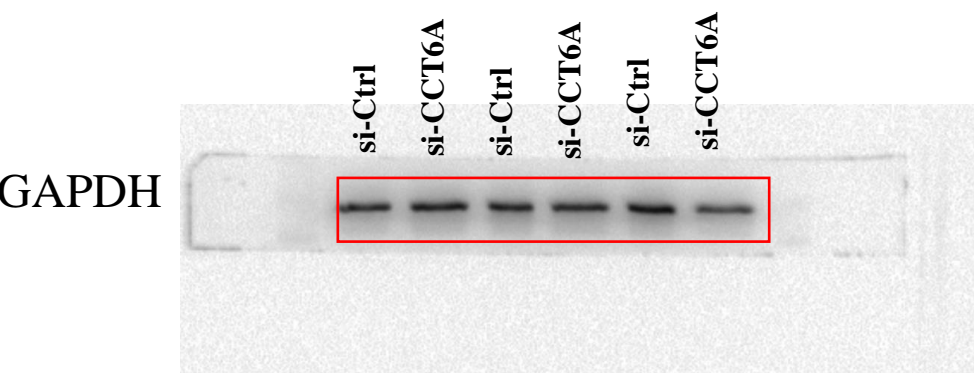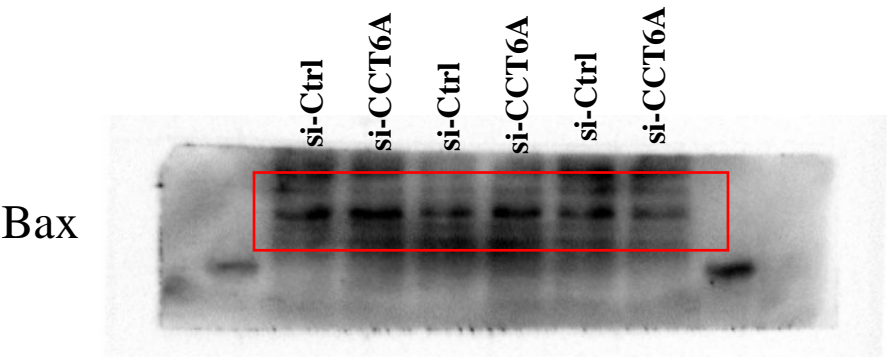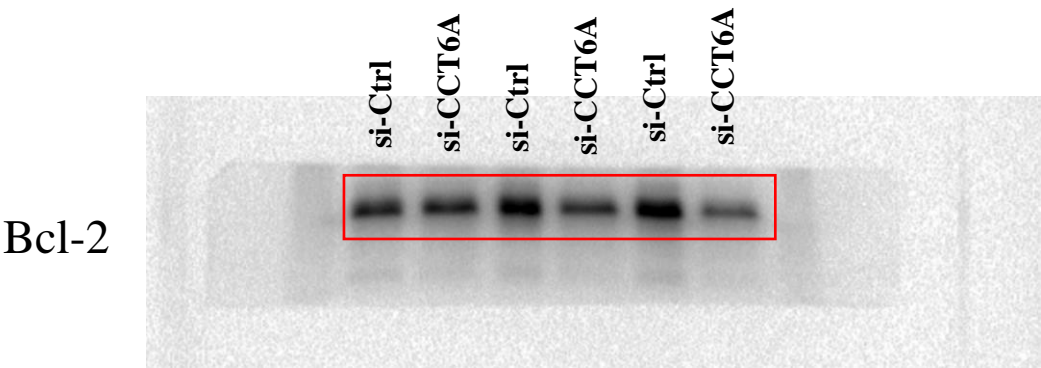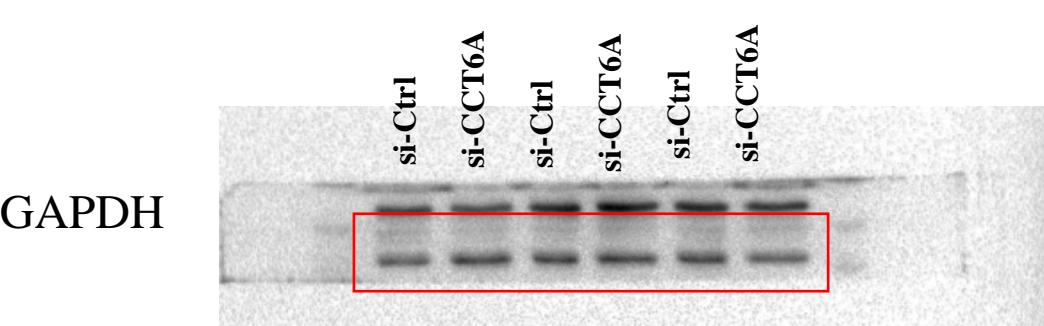

Figure 6

P-AKT

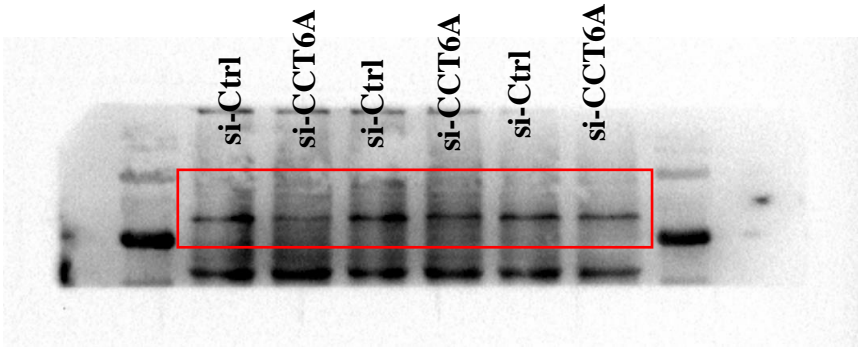

AKT

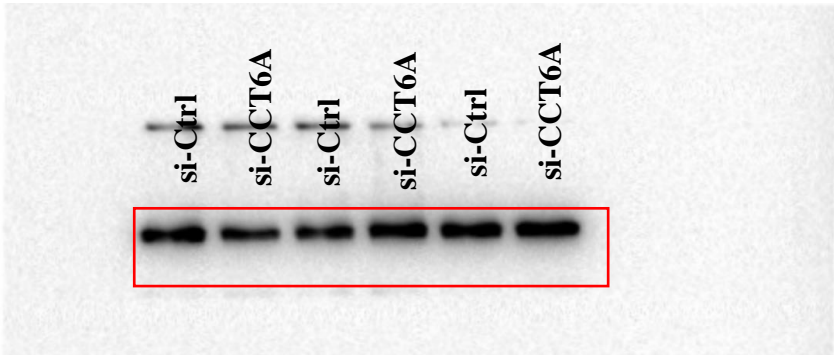

GAPDH

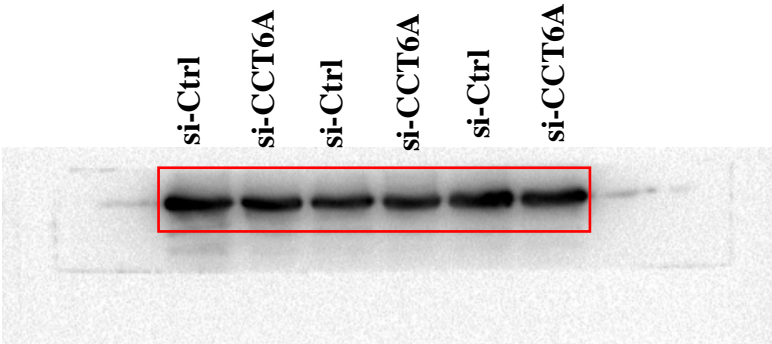

Supplement: S1 File — (PDF) [file pone.0279851.s002.pdf]
