# Supplementary material for: CCT6A knockdown suppresses osteosarcoma cell growth and Akt pathway activation in vitro
Source: PLoS One. 2022 Dec 30;17(12):e0279851. doi: 10.1371/journal.pone.0279851 (PMC9803215; doi:10.1371/journal.pone.0279851)
Supplement: Attachment — Submitted filename: Response to Reviewers-1201.doc [file pone.0279851.s003.doc]

Dear Dr Nigris and reviewers,

Great appreciate for all your valuable comments. We make a point-to-point response as following:

1. Please ensure that your manuscript meets PLOS ONE's style requirements, including those for file naming. The PLOS ONE style templates can be found at 
https://journals.plos.org/plosone/s/file?id=wjVg/PLOSOne_formatting_sample_main_body.pdf and 
https://journals.plos.org/plosone/s/file?id=ba62/PLOSOne_formatting_sample_title_authors_affiliations.pdf

Thanks. We revised the manuscript base on the templates. Please find the revised manuscript.

2. Thank you for stating the following in the Acknowledgments Section of your manuscript:

"This study was supported by National Natural Science Foundation of China (81803882), and Natural Science Foundation of Fujian Province (2020J06026)."

We note that you have provided funding information that is not currently declared in your Funding Statement. However, funding information should not appear in the Acknowledgments section or other areas of your manuscript. We will only publish funding information present in the Funding Statement section of the online submission form. 
Please remove any funding-related text from the manuscript and let us know how you would like to update your Funding Statement. Currently, your Funding Statement reads as follows:

"1、National Natural Science Foundation of China (81803882) . Funders:AS. He is Corresponding author. https://www.nsfc.gov.cn
2、 Natural Science Foundation of Fujian Province (2020J06026). Funders:AS. He is Corresponding author. http://xmgl.kjt.fujian.gov.cn/showLoginPage.do?type=fujian&loginflag=false"

Please include your amended statements within your cover letter; we will change the online submission form on your behalf.

Sure. We have removed the funding information from the Acknowledgments Section and updated in the Funding Statement, as well as stated the funding information in the cover letter

3. PLOS ONE now requires that authors provide the original uncropped and unadjusted images underlying all blot or gel results reported in a submission’s figures or Supporting Information files. This policy and the journal’s other requirements for blot/gel reporting and figure preparation are described in detail at https://journals.plos.org/plosone/s/figures#loc-blot-and-gel-reporting-requirements and https://journals.plos.org/plosone/s/figures#loc-preparing-figures-from-image-files.

Sure, we have added the PDF about all blot picture, please find the attachment.

1. Is the manuscript technically sound, and do the data support the conclusions?

The manuscript must describe a technically sound piece of scientific research with data that supports the conclusions. Experiments must have been conducted rigorously, with appropriate controls, replication, and sample sizes. The conclusions must be drawn appropriately based on the data presented.

All the data have three times repeat or six times repeat (CCK-8 assay), which were stated in figure legends sections within the revised manuscript. All data are represented as mean and SD. It makes sure the conclusions have drawn appropriately based on the data presented.

2. Has the statistical analysis been performed appropriately and rigorously?

All the statistical analyses were conducted using SPSS statistical software (version: 22.0). Data were presented as the mean ± standard deviation of at least three independent experiments. The difference between two groups were tested using the independent Student's t test, and among or more than 3 groups were analyzed using one-way ANOVA. P < 0.05 was considered significant.

3. Have the authors made all data underlying the findings in their manuscript fully available?

The [PLOS Data policy](http://www.plosone.org/static/policies.action#sharing) requires authors to make all data underlying the findings described in their manuscript fully available without restriction, with rare exception (please refer to the Data Availability Statement in the manuscript PDF file). The data should be provided as part of the manuscript or its supporting information, or deposited to a public repository. For example, in addition to summary statistics, the data points behind means, medians and variance measures should be available. If there are restrictions on publicly sharing data—e.g. participant privacy or use of data from a third party—those must be specified.

Sure, we provided the original data, including western blot picture and others, please find the attachment.

4. Is the manuscript presented in an intelligible fashion and written in standard English?

PLOS ONE does not copyedit accepted manuscripts, so the language in submitted articles must be clear, correct, and unambiguous. Any typographical or grammatical errors should be corrected at revision, so please note any specific errors here.

Thanks. The manuscript has been carefully reviewed by an experienced editor whose first language is English and who specializes in editing papers written by scientists whose native language is not English. Please find the attachment about CERTIFICATE OF EDITING.

Reviewer #1: The data do not support all the findings fully. Several additional experiments need.

1 in the abstract section there are several grammatica errors and repetitions

Thanks. Same as above, the manuscript has been carefully reviewed by an experienced editor whose first language is English and who specializes in editing papers written by scientists whose native language is not English. Please find the attachment about CERTIFICATE OF EDITING.

2 R2 wich kind of database is? Only two normal tissue and ten tumors were analyzed?

R2 Genomics Analysis and Visualization Platform, is an online datamining and discovery platform designed to assist the bio-medical researchers with limited to no Bioinformatics skills to perform data science tasks in the omics field. After double check, we only found two normal tissue and ten tumors in the R2 website. Due to the limited number of both normal and tumor tissues, we will collect the more samples for verification of CCT6A expression in our recently study.

3 How the authors giustificate a different effects of different clones if they express the same amount of residual CCT6A protein?

In Western-blot assay, BCA assay was performed according to the manufacturer's instructions to detect the total protein concentration. 50 μg of the total protein (all of samples with same protein concentration) was loaded and separated. Moreover, we also calculated the quantification of the protein expression using ImageLab software which supporting the different expression of CCT6A. We could make sure the protein expression of CCT6A in the same protein concentration.

4 Wich clones was selected? Plaese indícate in figure 3 and in figure 4.

Due to its prominent suppression of CCT6A protein expression and cell growth, si-CCT6A-3 was selected for further evaluation as si-CCT6A, which were indicated the figure legends section.

5 In figure 5 the authors evaluated apoptosys by facs. How they say the apoptosys rate was sixty %?

The cells in M1 phase were recognized the total Annexin V positive staining (about 60% in si-CCT6A group).

6 Counts on y axe is not a % of cells.

Thank you very much. We calculated the percentage of cells in different phases based on the counts in the export data from FACS. So we presented the percentage of cells on y axe.

7 Could the authors explain the pleiotropic role of CCT6A in transcriptional regiulation of several genes.

Our results shown that CCT6A knockdown decreased the expression of phosphorylation of Akt, it had only minor effects on the total expression of Akt. This suggests that suppression of the Akt phosphorylation might be among the underlying mechanisms behind the suppression of OS cell growth and the induction of apoptosis by CCT6A knockdown.

Discussion

In discussion section, please at line 245 also consider the effects of microenviroment in osteosarcoma progression in particular of immune components. See Mosca et all 2022.

Our study has some potential limitations, because we focused on the role of CCT6A in osteosarcoma cells without considering its effect on the tumor microenvironment and immune components. Further study, we will investigate that CCT6A participates

in both microenvironment and adaptive immune responses in OS cells.
